# Engaging Patient Advisory Boards of African American Community Members with Type 2 Diabetes in Implementing and Refining a Peer-Led Medication Adherence Intervention

**DOI:** 10.3390/pharmacy10020037

**Published:** 2022-03-10

**Authors:** Martha A. Maurer, Olayinka O. Shiyanbola, Mattigan L. Mott, Julia Means

**Affiliations:** 1 Sonderegger Research Center for Improved Medication Outcomes, School of Pharmacy, University of Wisconsin-Madison School of Pharmacy, Madison, WI 53705, USA; mamaurer@wisc.edu; 2 Division of Social and Administrative Sciences, School of Pharmacy, University of Wisconsin-Madison, Madison, WI 53705, USA; 3 School of Nursing, University of Wisconsin-Madison, Madison, WI 53705, USA; mlmott2@wisc.edu; 4 Ebenezer Ministry & Family Worship Center, Milwaukee, WI 53212, USA; meansju@aol.com

**Keywords:** type 2 diabetes, medicine use, adherence, community-engaged, peer support

## Abstract

African Americans are more likely than non-Hispanic whites to be diagnosed with and die from diabetes. A contributing factor to these health disparities is African Americans’ poor diabetes medication adherence that is due in part to sociocultural barriers (e.g., medicine and illness misperceptions), which negatively affect diabetes management. In our prior work, we engaged with community stakeholders to develop and test a brief version of a culturally adapted intervention to address these barriers to medication adherence. The objective of this study was to elicit feedback to inform the refinement of the full 8-week intervention. We utilized a community-engaged study design to conduct a series of meetings with two cohorts of patient advisory boards of African Americans with type 2 diabetes who were adherent to their diabetes medicines (i.e., peer ambassadors). In total, 15 peer ambassadors were paired with 21 African American participants (i.e., peer buddies) to provide specific intervention support as peers and serve in an advisory role as a board member. Data were collected during nine board meetings with the patient stakeholders. A qualitative thematic analysis of the data was conducted to synthesize the findings. Feedback from the patient advisory board contributed to refining the intervention in the immediate-term, short-term, and long-term. The inclusion of African American community members living with type 2 diabetes on the advisory board contributed to further tailoring the intervention to the specific needs of African Americans with type 2 diabetes in the community.

## 1. Introduction

Engaging patients and community members as stakeholders in research heightens the importance of the study for the target population and strengthens the quality of the research [1]. This type of engagement in the research process has the potential to: uncover new research questions; contribute a community perspective to inform the design of recruitment, retention, and research protocols, tailor interventions to the population of interest, and improve the dissemination of study findings to the community [1,2].

Using a community-engaged approach is particularly important for research focused on addressing health disparities experienced by underserved/marginalized populations because it facilitates building trust by creating an equal partnership between the academic researchers and the community and emphasizing a two-way exchange of knowledge [3,4,5]. An example is in mitigating diabetes disparities experienced by African Americans. Type 2 diabetes disproportionately affects African Americans, who are more than twice as likely as non-Hispanic whites to be diagnosed with type 2 diabetes and two to four times more likely to experience diabetes-related complications (e.g., kidney disease, amputations, and blindness) [6]. An important strategy for self-managing type 2 diabetes is taking medications to reduce blood glucose levels; however, African Americans have a 25% lower adherence to diabetes medicines than non-Hispanic whites [7,8]. 

Studies suggest that prior interventions to improve medication adherence among African Americans may not have been effective because they were not culturally tailored [9] or because of African Americans’ distrust of health care [10]. Therefore, to improve the relevance and acceptability of a medication adherence intervention for African Americans it is critical to engage them in all stages of designing and implementing the intervention. Ultimately, these efforts will contribute to advancing health equity in the broader African American community. 

We have used a community-engaged design throughout the entire iterative process to develop a culturally tailored intervention to provide African Americans with diabetes information, skill-development, and motivation to address psychosocial/sociocultural concerns and enhance diabetes medication adherence [11]. Initially, to inform the intervention components, we conducted focus groups with 40 African Americans with type 2 diabetes to identify their priority concerns related to medication adherence and diabetes self-management. Participants perceived that improving medication adherence required a peer to discuss beliefs and misperceptions about diabetes and diabetes medicines, including the necessity of medicines, and to support their self-confidence in diabetes self-management and control via positive empowerment [12]. Providing race-congruent support from someone with type 2 diabetes was critical along with group educational sessions. 

Subsequently, we developed a brief 3-week intervention that was tailored according to the feedback from African American community members. It involved peer support and group sessions as recommended by the focus groups [12,13]. Additionally, the intervention addressed the specific patient-related psychosocial and sociocultural barriers related to medication adherence that were raised by the African American community members. We again used a similar community-engaged approach to implement and refine the brief intervention [14,15]. We convened a patient advisory board of African American community members with type 2 diabetes who were adherent to their diabetes medications to provide feedback about the intervention during group meetings. The board members also served as peer ambassadors (PAs), African American community members who were adherent to their medications were matched with African Americans with type 2 diabetes who were nonadherent to their diabetes medicines (peer buddies - PBs) to deliver the peer support component of the intervention. We incorporated the feedback from the board and made changes when initially designing the full 8-week intervention. For example, we added a provider-led education session, expanded the number of phone calls to address sociocultural factors affecting adherence among African Americans and redesigned the logo for the program to better reflect peer support. The objective of this paper is to describe our approach to engaging with the African American community to implement, elicit feedback about, and refine the full 8-week peer-led medication adherence intervention.

## 2. Materials and Methods

### 2.1. Design

We utilized a community-engaged approach working with two patient stakeholder boards in different cities comprised of PAs to implement, elicit feedback about, and refine the Peers Supporting Health Literacy, Self-efficacy, Self-Advocacy, and Adherence (Peers LEAD) intervention.

### 2.2. The Intervention

The focus of this study was not on the delivery of the intervention; however, details of the intervention are provided as context for understanding the feedback PAs were providing. Peers LEAD is an 8-week educational–behavioral intervention that provides African Americans with culturally tailored diabetes and medication information, one-on-one peer support from African Americans with diabetes, and skills related to self-efficacy/provider communication to enhance medication adherence. This intervention builds on our prior developmental work in which we elicited input from African American community members in focus groups and then designed a brief 3-week intervention that incorporated the focus group findings from African American community members. Based on recommendations from the focus groups [12,13], the intervention includes peer support and group sessions and addresses the specific psychosocial and sociocultural barriers to medication adherence that were raised by the African American community members. In this study, we designed the remaining 5 weeks of the intervention then implemented and refined the full 8-week intervention. In Peers LEAD, African Americans with type 2 diabetes who were adherent to their diabetes medicines, PAs, were matched with African Americans with type 2 diabetes who were nonadherent to their diabetes medicines, PBs. 

The design of the intervention involved three 2 h group sessions (weeks 1, 2, and 8) and five phone calls between the PAs and PBs (weeks 3–7). The first group session was led by an African American diabetes educator and included a discussion of beliefs and misperceptions surrounding the cause of diabetes and beliefs about how diabetes affects one’s life, such as how it affects one’s emotions and how much control one feels one has over one’s diabetes. This culturally tailored content was based on our prior work exploring African Americans’ perceptions of diabetes and characterizing sociocultural factors that influenced their perceptions of the disease [12,13]. The week 2 group session was facilitated by a pharmacist who had experience working with individuals with type 2 diabetes in communities of color. In this session, the pharmacist guided a discussion focusing on beliefs about diabetes medicines, reasons for medicine nonadherence, and strategies for communicating with a pharmacist or other healthcare provider about diabetes medicines. Content for this session was tailored to explore and discuss African Americans’ reasons for not taking medicines and issues of provider mistrust/racial discrimination experiences in healthcare that impact the use of medicines in African Americans. 

Weeks 3–7 of the intervention involved the PAs making weekly calls to their PBs for a 15–30 min phone call. PAs guided the discussions and addressed five different intervention topics, including building self-confidence to take medicines correctly, coping with diabetes, fear, frustration, and emotional distress related to having diabetes, etc. Table 1 provides details of all intervention sessions and topics. In the final week of the intervention, a provider facilitated a group education session providing information about how diabetes works in the body, including potential complications, how diabetes medicines treat diabetes, and how patients can make the best use of doctor’s visits and approaches for communicating with their doctor. 

### 2.3. Stakeholders Involved in Community Engagement Process

The intervention implementation, collection of feedback from the community, and refinement of the intervention involved three groups of stakeholders: (1) academic collaborators (research team), (2) patient advisory boards composed of African Americans with type 2 diabetes, and (3) consultants from the Wisconsin Network for Research Support (WINRS) [16]. 

First, the interdisciplinary research team included experts in medication adherence, behavioral science, and chronic illness self-management among African Americans. The primary investigator (PI), who has expertise in illness perceptions, beliefs about medicines, and medication adherence among underserved populations, guided the intervention implementation and refinement. The academic collaborators contributed their expertise in addressing cultural health beliefs, experience in designing and implementing African American community-based interventions, and background in research on contextual challenges experienced by African American populations that make it difficult to manage diabetes. Monthly meetings were held with the PI and academic collaborators throughout the study to discuss how to engage the African American community in the intervention refinement and implementation and the best approach for incorporating the African American community’s perspective in the implementation and refinement of the intervention. 

Second, similar to our prior work, we again convened patient advisory boards (i.e., peer ambassador board—PAB). The inclusion criteria for PAs was self-identifying as African American or Black, being between the ages of 30 and 65 years old, taking one or more oral diabetes medicine, being diagnosed with type 2 diabetes for at least one year and the ability to communicate in English. Additionally, PAs had to be adherent to their diabetes medicines, which was assessed using the Adherence to Refills and Medication Scale—Diabetes (ARMS-D) [17]. To be eligible to be a PA, individuals had to receive a score of 11 on the self-reported ARMS-D scale, indicating optimal diabetes medication adherence. The PAB members engaged with the research team in an advisory role providing feedback on the intervention content and process based on their experience and knowledge. 

Third, as we have done in the past [15], we collaborated with WINRS, which is a patient and community engagement consultant group housed within the School of Nursing at the PI’s institution. Since 2010, WINRS staff have been consulting with researchers and facilitating stakeholder engagement, as well as training, planning, and facilitating more than 250 community-based advisory board meetings [18,19].

### 2.4. PAB Member Recruitment

Purposive sampling was used to recruit PAs. To identify potential PAs, we collaborated with community partners and individuals who served as PAs in our prior study [14,15]. This included inviting prior PAs to consider participation in the full 8-week intervention. Each potential PA was screened either on the phone or face to face to assess their eligibility based on the inclusion criteria and to gather information about informal criteria, such as their interest in participating in an advisory board, providing peer support, their diabetes experiences (e.g., motivation to take care of diabetes, level of confidence in taking diabetes medications), and their mentoring experience. PAs consented to be part of the program through a signed investigator responsibility form approved by the University’s Institutional Review Board.

### 2.5. Setting

The intervention and the community-engaged process to refine the intervention were conducted in 2019 and 2020 in two midwestern cities, involving two separate cohorts of PBs and PAs. 

### 2.6. PAB Member Role and Procedures

PAs served dual roles in this study: they delivered components of the intervention by providing support to the PBs and they served as PAB members providing feedback on the intervention design, refinement, and implementation. Details of their role in delivering the intervention are published elsewhere [20]. The PAB members engaged with the research team in an advisory role, providing feedback on the intervention content and process. Five 2-h stakeholder meetings (one for orientation and training, another for training, and three feedback meetings) were facilitated by the PI for each cohort of PAB members (Table 1 for further description). The meetings for the 2019 cohort were held in person at a community center at convenient times. The first two meetings for the 2020 cohort were held in person at convenient community-based locations, but the later meetings were held virtually using Webex due to COVID-19 pandemic restrictions on conducting face-to-face research. To compensate for their time and help with transportation costs, PAs were paid $25 per hour for each meeting they attended.

### 2.7. Engagement Procedures and Data Collection

The PI facilitated all PAB meetings using structured guides that were prepared in collaboration with WINRS. WINRS consulted with our research team on all PAB meetings and co-facilitated the board orientation and training meetings with the research team. Additionally, WINRS staff helped design program logistics, developed agendas and activities for meetings, drafted facilitator scripts/guides for each stakeholder meeting, and debriefed with the research team throughout the program to problem-solve emerging issues and identify strategies for improving the intervention processes and materials.

The meetings were held prior to, during, and after the 8-week intervention and each meeting focused on discussion topics related to the most recent intervention activities. Table 2 summarizes the meeting timeline and specific discussion topics. The process was iterative and bi-directional with the PI periodically sharing with the PABs how their feedback was being incorporated into intervention changes in real time, for the second cohort, and for the future. The research team recorded detailed notes on flip charts during the feedback meetings to facilitate discussion. These notes were then transcribed by a research team member to serve as record of the discussion. In addition, all meetings were audio-recorded and compared with hand-written notes to ensure the completeness of the data captured.

### 2.8. Data Analysis

After each PAB meeting, the research team shared the meeting documentation notes with WINRS and met in person to debrief and discuss. All feedback was considered, and a consensus was reached about whether the recommended changes were feasible to be implemented in real-time (i.e., for the second cohort in 2020) or for future studies or whether they warranted further consideration and exploration. When relevant, the research team also discussed the PAB feedback with the academic collaborators to get their expert input on how to best address the feedback and what implications it may have on the study design or methods. A study team researcher with expertise and experience in qualitative data analysis conducted a thematic analysis of the PAB meeting notes to synthesize the feedback into themes and sub-themes.

## 3. Results

In total, 15 African American PAs were paired with 21 PBs to provide specific intervention support as peers and serve in an advisory role as a board member. The 2019 and 2020 cohorts were comprised of 8 and 7 peer PAs, respectively. PAs (57 ± 7.5) were similar in age, had been diagnosed with diabetes for similar lengths of time (a mean of approximately 10 years), and were mostly female across both cohorts. Details of the intervention feasibility outcomes, including recruitment and retention of the PAs and PBs, are published elsewhere [20].

All PAs participated in a similar number of board meetings. Those in the 2019 cohort participated in five board meetings—all of which were held in person in the community. The PAs in the 2020 cohort participated in four meetings (Table 2), two of which were held in person and two of which were held virtually over a web-based virtual platform due to the COVID-19 pandemic.

The engagement with the PABs was meaningful and successful in accomplishing the aim of eliciting feedback about the intervention to inform refinements. The feedback provided by the PAs can be categorized into four key themes: (1) the training, resources, and tools provided to them to implement the intervention, (2) additions or modifications to the intervention education session content, (3) how the intervention was tailored for African Americans, and (4) barriers to and facilitators of engaging with the PBs. Table 3 summarizes the thematic analysis of the PAB member feedback related to refining the intervention.

Feedback from PAs had a significant impact and contributed to refining the intervention both in the immediate-term, short-term, and long-term. The PA feedback resulted in the research team making some immediate, real-time changes to the intervention, such as removing the icebreaker question from the agenda for subsequent virtual meetings and having the research team immediately respond to a request to provide PAs and PBs with additional help for navigating the technology for virtual meetings. In the short-term, some of the feedback from the 2019 cohort PAB was applied when implementing the intervention for the 2020 cohort. For example, the research team changed the checklist documents for the 2020 cohort, making two versions of the form that were less repetitive to better capture the evolution of the relationship and the research team changed the sequence of sessions for the 2020 cohort so that all three group sessions were conducted prior to the phone calls. Additionally, the research team is considering other PA input for future changes to the intervention. The research team has continued to refine the intervention by incorporating PAB feedback and planning a study to test the addition of a diabetes self-management program to Peers LEAD to address topics such as diet, exercise, and the addition of a community health worker to help address access to community resources. Some PAB feedback is still being considered by the research team.

## 4. Discussion

This study utilized a community-engaged collaborative approach involving patient stakeholders throughout the research process by directly engaging African American community members with diabetes who are successfully taking their medicines to utilize their experience, knowledge, and advice [3,4].

Overall, the engagement with the PAB was successful, which we attribute to several key factors. First, the academic study team collaboration with WINRS to develop the training and feedback meeting agendas/activities ensured the incorporation of strategies for effective engagement during meetings. All meetings began with an ice breaker exercise to allow the PAB members to get to know one another on a personal basis. Meetings also included reminders about respectful interaction with one another and an acknowledgement that all opinions and voices were welcome. Second, at each meeting the study team told the PAs how their contributions were making a difference in refinements that were being made to the intervention. This demonstrated the value of the PAs’ perspectives and was a recognition of how they were making a difference. Third, the structure and frequency of the PAB meetings provided an opportunity for PAs to build rapport and trust with each other, as well as with the PI and study team, which facilitated the development of an equitable partnership. Fourth, the study team supported PAs through weekly phone call check-ins to respond to questions and help to problem solve or address any concerns. This frequent, consistent, one-on-one communication may have enhanced the sustained involvement of PAs in the PAB. Lastly, the study team made efforts to accommodate PA’s practical needs by compensating them for their time, holding the meetings at a convenient time of day in their community, and providing diabetic-friendly meals. Many of these strategies are recommended best practices for successful engagement [19,21].

Despite these successes, we also encountered some challenges in our engagement efforts and learned some valuable lessons [22]. Some PAs who initially committed to being PAB members missed meetings due to personal matters or stepped away from the role part way through the study. To mitigate shortfalls in retaining the PAB members in the future, we learned to be clearer in our initial communications about the time commitment during the recruitment phase and to recruit more PAs than our ideal target, knowing that there may be some dropouts. Future studies will consider having trained PAs that are available to step into the role if needed. A unique aspect of this study was that our patient stakeholders, the PAs, served dual roles in the research process, implementing a portion of the intervention and contributing as a member of the PAB. A strength of this approach is that the PAs had first-hand experience with the intervention, giving them a unique and rich perspective. However, being so close to the intervention may have narrowly focused their perspectives, making it challenging to consider broader questions about the intervention and how it relates to healthcare systems or other diabetes self-management programs. We learned that including additional external perspectives on the intervention could further enhance the refinement of the intervention.

Another notable challenge was the emergence of COVID-19 in March 2020 and statewide stay-at-home orders that interrupted the 2020 PA cohort. This was particularly challenging considering the disproportionate number of COVID-19 hospitalizations and deaths in the African American community and the unknown effects on individuals with diabetes [23]. The cohort was initiated in February 2020 and initial meetings were held in person, but then abruptly shifted to a virtual web-based platform. While this resulted in a delay in the study and presented numerous challenges, we were able to elicit rich feedback related to COVID-19 from PAs who experienced the intervention both in-person and virtually. PAs recognized this was beyond the control of the study team. The research team will take this feedback into consideration when weighing the costs and benefits of holding group sessions in person or virtually. 

This study contributes to the community-engaged research literature by documenting our process of engaging with patient stakeholders and its impact on the refinement of a medication adherence intervention. While the approach of engaging with patient stakeholders in designing and conducting research has become more prevalent over the last decade, research focused on the engagement process and outcomes is a growing body of literature [22,24,25]. By reporting our experiences, we provide practical guidance and examples for successful community engagement, adding to the growing body of evidence for researchers [26].

Our findings also contribute to the field of community engagement in research focused on addressing health disparities experienced by underserved/marginalized populations [3,4]. We demonstrate how positive engagement and eliciting the perspective of patient stakeholders in the African American community validated certain intervention characteristics and provided support for retaining and enhancing those components. PAs in both cohorts acknowledged the cultural tailoring of the intervention for African Americans as a positive aspect of the intervention. The 2019 cohort PAB appreciated that the provider leading the group session was a person of color who had extensive experience working with the African American community and the 2020 cohort PAB reflected positively on the provider relating the science of diabetes to beliefs and myths that African Americans typically have about diabetes. In addition, the 2019 cohort PAB reported that they valued a program with all African American participants who have shared experiences with diabetes. They expressed that this facilitated building trust among the group and between the PAs and PBs. This feedback is particularly important as it suggests that the intervention was perceived by the PABs as relevant for African Americans, which is in line with the goal of the intervention to be culturally tailored. This represents the essence of how a community-engaged process to develop, implement, and refine an intervention can serve to advance work to address health disparities [4].

There were some study limitations. First, we did not use a structured process and/or validated tool to evaluate PA’s skills in communicating with and supporting PBs. PAs who met the inclusion criteria may not have been well-equipped to take on the role of providing peer support. Second, we assessed PA medication adherence using a self-report measure, which is not objective and may not accurately reflect the PA’s level of medication adherence. If a PA was not adherent to their diabetes medicines, they may have struggled to provide peer support and address misperceptions about illness and medicines with which PBs were struggling. Third, we changed our method of data collection from in-person for the 2019 cohort to online for the 2020 cohort due to the COVID-19 pandemic restrictions on meeting in person. Research comparing in-person and online qualitative data collection has found differences in the quantity of data collected via each method; however, some research suggests that the richness of the data is comparable across both methods [27]. Fourth, we focused solely on the perspectives of community members, the PAs, PBs, and African Americans with type 2 diabetes. However, there are other important perspectives that should be taken into consideration related to diabetes medication adherence programs, such as those of diabetes educators, providers, caregivers, friends/family members and community organizations, that we did not focus on as part of this study. Finally, given the similarity in ages between the PAs and PBs, there may be limited generalizability of the feedback about the intervention for younger or older African Americans.

## 5. Conclusions

In this study, three stakeholder groups (i.e., academic collaborators, consultants on community-engaged research and a patient advisory board of African American community members) collaborated to implement and refine a peer-led medication adherence intervention by providing feedback throughout the process. Feedback received from the PAB was considered by the research team and consultants and many changes were made to the intervention either immediately or in the short-term. In addition, the research team plans to implement several changes moving forward and continues to consider others for potential future changes or additions to the intervention. The inclusion of African American community members living with type 2 diabetes as the advisory board contributed to further tailoring the intervention to the specific needs of African Americans with type 2 diabetes in the community. We continued to engage with the African American community for a recently completed study testing the next iteration of the intervention that pairs the culturally tailored peer support and group education sessions with an evidence-based diabetes self-management program for a new cohort of PBs and PAs. Future studies should engage with underserved/marginalized communities when designing, implementing, and refining an intervention to ensure that interventions are aligned with and address the needs of the community.

## Figures and Tables

**Table 1:** Peers LEAD 8-week intervention.

Week	Intervention Component	Details of the Intervention Delivery
Week 1	Target negative illness beliefs with a focus on the cause and consequence of diabetes.	Group session with PAs and PBs led by a diabetes educator.
Week 2	Reframe medication beliefs to decrease medication concerns and increase the necessity of medicines. Address reasons for nonadherence. Discuss pharmacist as a resource and strategies for communicating with pharmacists.	Group session with PAs and PBs led by a pharmacist.
Week 3	Discuss self-efficacy and coping with diabetes.	One-on-one phone call (15–30 min) between PA and PB dyads.
Week 4	Provide support for addressing fear, frustration, and emotional distress.	One-on-one phone call (15–30 min) between PA and PB dyads.
Week 5	Discuss self-advocacy in provider communication and relationship building.	One-on-one phone call (15–30 min) between PA and PB dyads.
Week 6	Discuss family/community bonding and maintaining cultural experiences.	One-on-one phone call (15–30 min) between PA and PB dyads.
Week 7	Discuss setting goals related to taking diabetes medicines to continue self-managing after the program ends.	One-on-one phone call (15–30 min) between PA and PB dyads.
Week 8	Provide information about how diabetes works in the body. Discuss how to make the most of doctor’s appointments and approaches to talking with providers.	Group session with PAs and PBs led by a provider.

**Table 2:** PAB meeting timeline and discussion topics.

Meeting	Timeframe	Discussion Topics
PA Orientation	2–3 weeks prior to beginning of 8-week intervention	Overall project goal, roles of all project stakeholders, demonstration of PA role in listening and supporting a PB.
PA Training	1–2 weeks prior to beginning of 8-week intervention	Preparation for phone calls with PBs, detailed phone call suggestions, at-a-glance telephone call guide
First feedback meeting	Week 5 of the 8-week intervention (after initial group sessions and three phone calls with peer buddies)	Feedback about the group education sessions: session content (whether the information was useful and interesting, as well as what information was not included and should be added) and session format (length, mode—mix of discussion, lecture, and question time).Feedback about the initial three phone calls with their PBs: what worked well and could have been better during the phone calls.
Second feedback meeting	Immediately after the completion of the 8-week intervention	PI shared examples of how PAs were making a difference and what intervention changes were being made based on their feedback; PA feedback about the provider group education session: session content and format; PA reflections on final phone calls with PBs: what worked well and what could have been better; discussion of tips for future PAs on preparing for phone calls; feedback about training/tools that were discussed at orientation and training meetings; PI collected feedback about resources provided to PAs and recommendations for additional resources.
Third feedback meeting ^	2 weeks after the completion of the 8-week intervention	PI shared examples of how PAs were making a difference based on their feedback from the second feedback meeting; PA feedback on the process and materials for recruiting future PAs and suggestions for additional materials; PA discussed experience with other diabetes management programs; PI showed template for how a PA could include their membership in the board as part of their resume or social media platform.

^ A third feedback meeting was not conducted for the 2020 cohort due to COVID-19 pandemic restrictions.

**Table 3:** Summary of PAB feedback about intervention refinement.

Theme/Subthemes	Representative Examples of PAB Feedback
**Theme: PA training, resources, and tools**	
***Subthemes***	
Need for additional PA training and resources.	Suggested including additional information and resources in program manuals (e.g., information about diabetes, mental health, and additional community resources) (2019 and 2020).
Resources/tools provided to PAs useful for facilitating peer-led phone calls.	Checking off topics/making notes on telephone call guide prior to calls facilitates the PA being prepared for call (2020).
**Theme: Education session content**	
***Subthemes***	
Additions to education session content.	Suggested adding information about the five to ten most common diabetes medications such as metformin (2019).
Modifying sequence of presenting content.	Suggested holding all group sessions prior to the phone calls and having the provider present first to lay foundation about diabetes (2019).
**Theme: Tailoring intervention for African Americans**	
***Subthemes***	
Relating content to African Americans.	Appreciated that the provider shared the science of diabetes in layman terms and related it to beliefs/myths that African Americans often have about the disease (2020).
Importance of education session experts’ experience with African American community.	Appreciated that the provider was a person of color who had extensive experience working with the African American community (2019).
Unique value of Peers LEAD compared with other diabetes self-management programs.	Having all African American participants allowed for building trust; liked that everyone had type 2 diabetes – shared peer experiences; appreciated the one-on-one pairing with the PBs (2019).
**Theme: Engagement with PBs**	
***Subthemes***	
Difficult to engage meaningfully in virtual meetings.	Virtual format (due to COVID-19) was a barrier for PBs to feel comfortable and engaged (2020).
Meeting format facilitated relationship building with PBs.	In-person, small group discussions were a great opportunity to build relationships with PBs (2019).
Improving relationship building with PBs.	Suggested adding more time to joint orientation meeting for PAs/PBs to get to know each other (2019).
